# Development and Interpretation of New Sediment Rating Curve Considering the Effect of Vegetation Cover for Asian Basins

**DOI:** 10.1155/2013/154375

**Published:** 2013-12-24

**Authors:** Jie Wang, Hiroshi Ishidaira, Wenchao Sun, Shaowei Ning

**Affiliations:** ^1^Interdisciplinary Graduate School of Medicine and Engineering, University of Yamanashi, Kofu, Yamanashi 400-8511, Japan; ^2^College of Water Sciences, Beijing Normal University, Xinjiekouwai Street 19, Beijing 100875, China

## Abstract

Suspended sediment concentration of a river can provide very important perspective on erosion or soil loss of one river basin ecosystem. The changes of land use and land cover, such as deforestation or afforestation, affect sediment yield process of a catchment through changing the hydrological cycle of the area. A sediment rating curve can describe the average relation between discharge and suspended sediment concentration for a certain location. However, the sediment load of a river is likely to be undersimulated from water discharge using least squares regression of log-transformed variables and the sediment rating curve does not consider temporal changes of vegetation cover. The Normalized Difference Vegetation Index (NDVI) can well be used to analyze the status of the vegetation cover well. Thus long time monthly NDVI data was used to detect vegetation change in the past 19 years in this study. Then monthly suspended sediment concentration and discharge from 1988 to 2006 in Laichau station were used to develop one new sediment rating curve and were validated in other Asian basins. The new sediment model can describe the relationship among sediment yield, streamflow, and vegetation cover, which can be the basis for soil conservation and sustainable ecosystem management.

## 1. Introduction

The issue of soil erosion and sediment load of the watershed is one of the hot spots, which currently causes the global widespread attention. River sediment load is affected by climate change land cover change within its drainage basin in an integrated way [1, 2]. With the increase of population and rapid development of economics and society, human activities have been seriously affecting the watershed land cover, which in turn affect sediment yield response of a catchment through modifying the surface gradient, surface roughness, and soil erodibility [3]. In addition, sediment load also responds to climate variability which could not be negligible. In the face of intricate impacts from recent environmental changes, it is becoming a more complicated problem to calculate and evaluate sediment loads especially for basins with limited data.

In general, all kinds of sediment yield/load calculation models fall into three main categories: empirical or statistical, conceptual, and physical-based models. Merritt et al. [4] summarized these models in terms of their classification, scales of application, and input data requirements and concluded that model components generally contain a mix of empirical, conceptual, and physics-based algorithms. However, the distinction between models is not sharp and therefore can be somewhat subjective. it is difficult to sort these models exactly according to physical processes and model algorithms. They are likely to contain a mix of modules from each of these categories [4]. Because of this, we would like to classify all into models with more parameters and models with less parameter by amount of model parameters or input requirements. Among models with more parameters, physical-based models accounts for the main part. This kind of model could be universally used to predict sediment yield/load of a watershed and to provide an indication of the qualitative and quantitative effects of land use changes or climate change. However, it is always very difficult to determine value of parameters especially at describing the complex process of erosion and sediment yield. Due to the requirement that parameter values are determined through calibration against observed data, these models tend to suffer from problems associated with the identifiability of their parameter values and nonuniqueness of “best fit” solutions [4–6]. In addition, models with more parameters generally require a lot of detailed information including hydrological, hydraulic and geological characteristics of the river basin, as well as the sediment characteristics themself. Preparation of such dataset will be difficult and costly.

As for models with less parameters, sediment rating curves [7] representing a linear or nonlinear functional relationship that relates suspended sediment concentration/load to streamflow are one main type. Among all sediment rating curves, one power functional relationship is the most common one which describes the average relation between streamflow (*Q*) and suspended sediment concentration (SSC) for a certain location [8]. Because measuring the sediment flux on a river is a time-consuming and expensive operation, the sediment rating curve provides one considerable way to estimate sediment flux in the routine measurement of the flow in our rivers. As you might imagine, measuring the streamflow is not less time-consuming and expensive and for these same reasons we make considerable use of the suspended sediment rating curve. In the absence of actual suspended sediment concentration measurements, hydrologists have used sediment rating curves to estimate suspended sediment concentrations [8]. Despite of increasing interest in sediment rating curves application over recent decades [7–10], it is still imperfectly understood especially in forest watersheds. In addition, it is too simple without considering temporal dynamic changes of vegetation cover [11]. However, different vegetation cover should have different effect on soil erosion production by modifying soil erodibility and transport capacity by slowing flow through friction losses [12].

Summarily, for basins without enough input data for physical-based sediment yield/load calculation models, sediment rating curve provides one opportunity to evaluate sediment load. And on purpose of this, the objective of this study is that one new sediment rating curve with few parameters considering the effect of vegetation cover with limitation information will be developed to calculate land cover change effect on the sediment load in this study. The Normalized Difference Vegetation Index (NDVI) can well be used to analyze the status of the vegetation coverage well. Thus long time monthly NDVI data was used to detect vegetation change in the past 19 years in this study. And monthly suspended sediment concentration and discharge from 1988 to 2006 at Laichau station (Da River Basin) were used to develop and interpret one new sediment rating curve. Compared with the common sediment rating curve, the new curve can simulate the suspended sediment concentration much better in the Da River Basin. In addition, we also compared new sediment rating curve simulation result with SWAT model simulation result. Finally, we also applied new sediment rating in another two basins and got promising results. The new curve can describe the relationship among sediment yield, streamflow, and vegetation cover, which can be the basis for soil conservation and sustainable ecosystem management.

## 2. Dataset and Methodology

### 2.1. Study Area and Data Description

**Figure 1:**
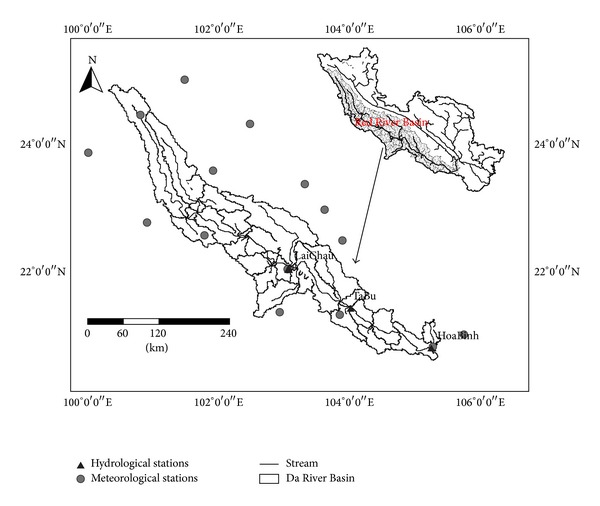
Location of the Da River Basin and the meteorological stations.

The Da River Basin (DRB) located in humid region is the biggest branch of the Red River which gets its name from the reddish-brown color caused by its high sediment load rich in iron dioxide. The DRB drains 55,000 km^2^ and originates in Yunnan Province, China (Figure 1). The river cross-sections are narrow, with a steep slope of 0.37. The annual mean runoff is about 1168 m^3^/s from 1988 to 2004 at Laichau station, associated with the total annual sediment load of about 40.1 × 10^6^ t/yr. The Da River Basin is characterized by a tropical monsoonal. Summer season is warm and humid, whereas the winter season is cool and dry. The annual mean rainfall is about 1320 mm for the Da River Basin, 85% of which falls during wet rainy season [13]. The HoaBinh reservoir, located on the downstream of Laichau station, is one of the largest (*V* = 9.5 km^3^) dams in South-East Asia. It was completely finished in 1993, with the main purpose of flood control, irrigation, and hydropower generation. Forest cover spreads over almost half of the total Da River Basin, in which evergreen broadleaf forest is the dominant vegetation type. Deforestation has become an issue of increasing concern in Red River Basin [13].

**Figure 2:**
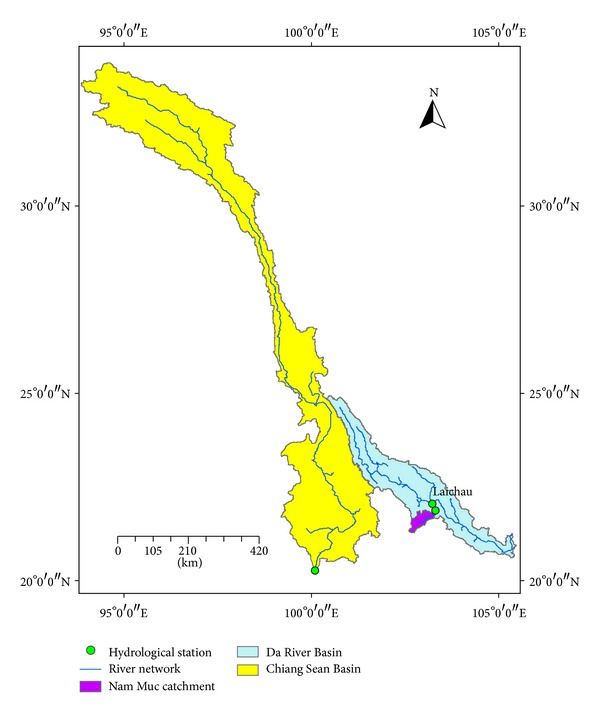
Location of three target basins.

In addition to Da River Basin, another two target basins in East-south Asian were selected to validate new sediment rating curve: Chiang Sean Basin in the upper part of Mekong River Basin and Nam Muc Basin in Red River Basin (Figure 2). The basic characters of basins are showed in Table 1.

In addition to the basic hydrological data showed in Table 1, other spatial datasets are also used for our research. There are 16 meteorological stations located in or around Da River Basin, which are spatially well distributed to reflect the characteristics of regional climate. Metrohydrologic data is from the China Meteorological Data Sharing Service and Vietnam Academy of Science and Technology, which has been checked by the primary quality control. SRTM 90 m DEM data is provided by the CIAT-CSI (http://srtm.csi.cgiar.org/). Global 1 km Land Cover data in the year of 1992 obtained from the US Geological Survey's National Center for Earth Resources Observation Science is also employed in the study. The GIMMS NDVI dataset [14] including a 25-year period from 1981 to 2006 is used to analyze the vegetation changes in a long time period for the study area.

### 2.2. Basic Research Framework

**Figure 3:**
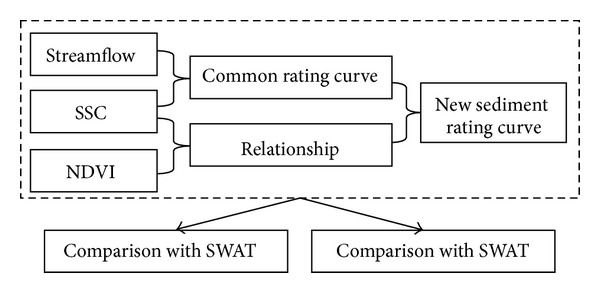
The research framework for developing new sediment rating curve.

According to basin research flowchart (Figure 3), we would firstly check the limitation of common rating curve and find out the inverse relationship between vegetation cover change and SSC in the Da River Basin. Then new sediment rating curve was developed based on this inverse relationship and the limitation of common rating curve in Da River Basin. In addition, result of new sediment rating curve was compared with one physical sediment calculation model. Finally, new sediment rating curve was validated in other East-south Asian basins.

### 2.3. Common Sediment Rating Curve

Among all sediment rating curves, one power functional relationship is most common one which describes the average relation between streamflow (*Q*) and suspended sediment concentration (SSC) for a certain location [8]. Consider the following:

(1) $$\begin{array}{c} \text{SSC}=aQ^{b},\\ \text{SL}=Q\times \text{SSC}, \end{array}$$

in which *Q* (m^3^/s) is discharge, SSC (g/m^3^) is suspended sediment concentration, and SL (g/s) is sediment load.

As reviewed before, we have already known that common sediment rating curve without considering temporal dynamic changes of vegetation cover is not so reasonable and generally could not get agreement simulation results. In addition, the physical-based sediment yield calculation model can give better results. However, these kinds of models have more parameters to identify and need more input dataset.

### 2.4. SWAT Model

The Soil and Water Assessment Tool (SWAT model) is considered as one of the popular physical-based models for calculating discharge, sediment load in large complex watersheds with varying soils, land use, and management conditions. In this study, SWAT model will be compared with new sediment rating curve.

SWAT is a continuous, long-term, and distributed parameter model based on water balance, designed to evaluate the impact of climate and land use change on the hydrology and sediment transport in watersheds. The relationship between input and output variables is described by regression equations. The SWAT model integrates all relevant ecohydrological processes including water flow, nutrient transport and turn-over, vegetation growth, and land use and water management at the subbasin scale. Consequently, the watershed is subdivided into subbasins based on the number of tributaries. Size and number of subbasins are variable, depending on stream network and size of the entire watershed. Subbasins are further disaggregated into classes of Hydrological Response Units (HRU), whereby each unique combination of the underlying geographical maps (soils, land use, etc.) forms one class. HRU are the spatial unit where the vertical flows of water and nutrients are calculated, which are then aggregated and summed for each subbasin. Water and material from HRU in subwatersheds are routed to the subwatershed outlet. The HRU in SWAT are spatially implicit, their exact position in the landscape is unknown, and it might be that the same HRU cover different locations in a sub-basin. The water balance for each HRU is represented by the four storages snow, soil profile, shallow aquifer, and deep aquifer. The soil profile can be subdivided into up to ten soil layers. Soil water processes include evaporation, surface runoff, infiltration, plant uptake, lateral flow, and percolation to lower layers [15].

The sediment from sheet erosion for each HRU is calculated using the Modified Universal Soil Loss Equation (MUSLE) [16]. Details of the USLE equation factors can be found in Neitsch et al. [15]. The sediment concentration is obtained from the sediment yield, which corresponds to flow volume within the channel on a given day. The transport of sediment in the channel is controlled by simultaneous operation of two processes: deposition and degradation. Whether channel deposition or channel degradation occurs depends on the sediment loads from the upland areas and the transport capacity of the channel network. If the sediment load in a channel segment is larger than its sediment transport capacity, channel deposition will be the dominant process. Otherwise, channel degradation occurs over the channel segment. Theory and details of hydrological and sediment transport processes integrated in SWAT model are available online in SWAT documentation (http://swatmodel.tamu.edu/).

### 2.5. Model Validation Method

Following recommendations [17], four statistics are used to indicate the accuracy of SWAT model and sediment rating curves: coefficient of determination (*R* ^2^), Nash-Sutcliffe efficiency (NSE), percent bias (PBIAS) and the mean absolute error (MAE). The use of these statistics is to provide a more comprehensive evaluation of the model performance. Consider the following:

(2) NSE=1−∑i=1n(Qobsi−Qsimi)2∑i=1n(Qobsi−Qobs−)2,R2=∑i=1n(Qobsi−Qobs¯)(Qsimi−Qsim¯)∑i=1n(Qobsi−Qobs¯)∑i=1n(Qsimi−Qsim¯),PBIAS=|Qsim¯−Qobs¯|  Qobs−∗100%,MAE=|Qsim¯−Qobs¯|,

where *Q*_sim_ is simulated discharge, *Q*_obs_ is observed discharge, $\bar{Q_{\text{sim}}}$ is average simulated discharge, and $\bar{Q_{\text{obs}}}$ is average observed discharge.

## 3. Results and Discussions

### 3.1. Limitation of Common Sediment Rating Curve

**Figure 4:**
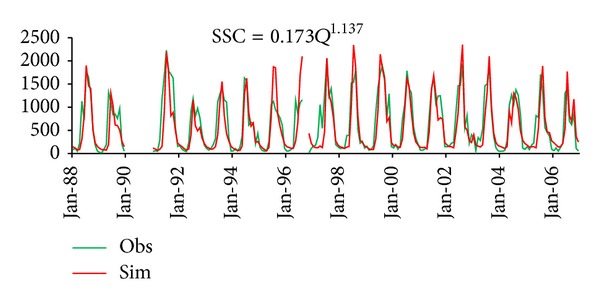
Comparison of monthly observed SSC and simulated SSC from common sediment rating curve.

In order to check out the limitation of common sediment rating curve, common sediment rating curve was first applied in Da River Basin (Figure 4). The mean absolute error (MAE) between simulated and observed SSC was also calculated for high and low values, as shown in Table 2. Results showed that the common sediment rating curve method underpredicted low and whole SSC and overpredicted high SSC values in Da River Basin, which kept the same shortage with some previous researches.

### 3.2. Relationship between NDVI and SSC

**Figure 5:**
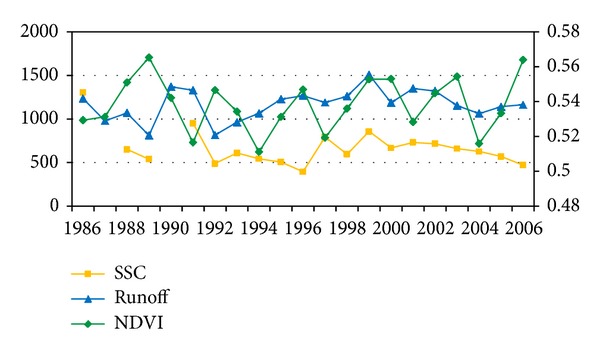
Comparison among annual SSC, runoff and NDVI in Da River Basin.

Different vegetation cover should have different effect on soil erosion production and transport capacity by slowing flow through friction losses. In the wet season and wet year, vegetation cover is better and the soil erosion production and transport capacity should be lower. The facts in the dry season are just the opposite. In order to check out the relationship among runoff, NDVI, and SSC, changes of annual time series were showed in Figure 5; the similar change trend between SSC and runoff indicates that SSC is mainly controlled by runoff. However, some years show inverse trend, such as 1996, 2005, and 2006. In these three years, SSC shows lower values even though runoff became higher because the higher NDVI could reduce the soil erosion production and transport capacity. In addition, the negative correlation coefficient between SSC and NDVI (−0.31) also makes the inverse relation more clear between NDVI and SSC. This inverse relationship between NDVI and SSC could also explain the reason of the limitation of common rating curve simulation.

### 3.3. New Sediment Rating Curve Development

Previous studies combining results from different watersheds provided physical interpretations of the two parameters in the common sediment rating curve [7, 8, 18, 19]. The common sediment rating curve coefficient “*a*” may give information on the soil erodibility and transport capacity for the whole basin, and “*b*” may represent the erosive power of the river and its sediment transport capacity. As a result, vegetation cover should only affect coefficient “*a*” in a river basin. Based on the inverse relationship between NDVI and SSC, we tried to add vegetation cover information (NDVI) into coefficient “*a*” and carried out the three following types of new sediment rating curves:

(3) $$\begin{array}{c} \text{SSC}=a\left(c-\text{NDVI}\right)Q^{b},\\ \text{SSC}=\frac{a}{\left(c+{\text{NDVI}}^{d}\right)Q^{b}},\\ \text{SSC}=a\left(1-{\text{NDVI}}^{c}\right)Q^{b}, \end{array}$$

in which *Q* (m^3^/s) is discharge, SSC (g/m^3^) is suspended sediment concentration and Parameter of *a*, *b*, *c* and *d* are determined from data via least squares method.

Then three calibrated new sediment rating curves were developed for Da River Basin as follows:

(4) $$\begin{array}{c} A\text{:}\;\;\text{SSC}=0.17\left(1.75-\text{NDVI}\right)Q^{1.137},\\ B\text{:}\;\;\text{SSC}=\frac{0.28}{\left(1.3+{\text{NDVI}}^{4.5}\right)Q^{1.137}},\\ C\text{:}\;\;\text{SSC}=0.234\left(1-{\text{NDVI}}^{5.3}\right)Q^{1.137}. \end{array}$$

As shown in Table 3, three new sediment rating curves also underpredicted low and average SSC and overpredicted high SSC; however, all these new rating curves reduced the mean difference between simulated SSC and observed SSC in various degrees. In addition, all the coefficients of determination of three new sediment rating curves are also higher than common one, which indicates that vegetation cover information could improve the common sediment rating curve in Da River Basin. Among the three new sediment rating curves, type c could improve common one most and get the best simulation results. Consequently, type c is considered the most agreement sediment rating curve in Da River Basin, which is the new sediment rating curve.

### 3.4. Comparison with SWAT Model

**Figure 6:**
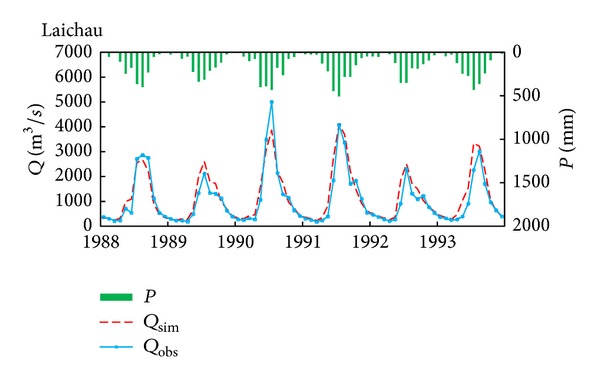
Comparison of observed and simulated monthly streamflow in the DRB (Laichau station).

**Figure 7:**
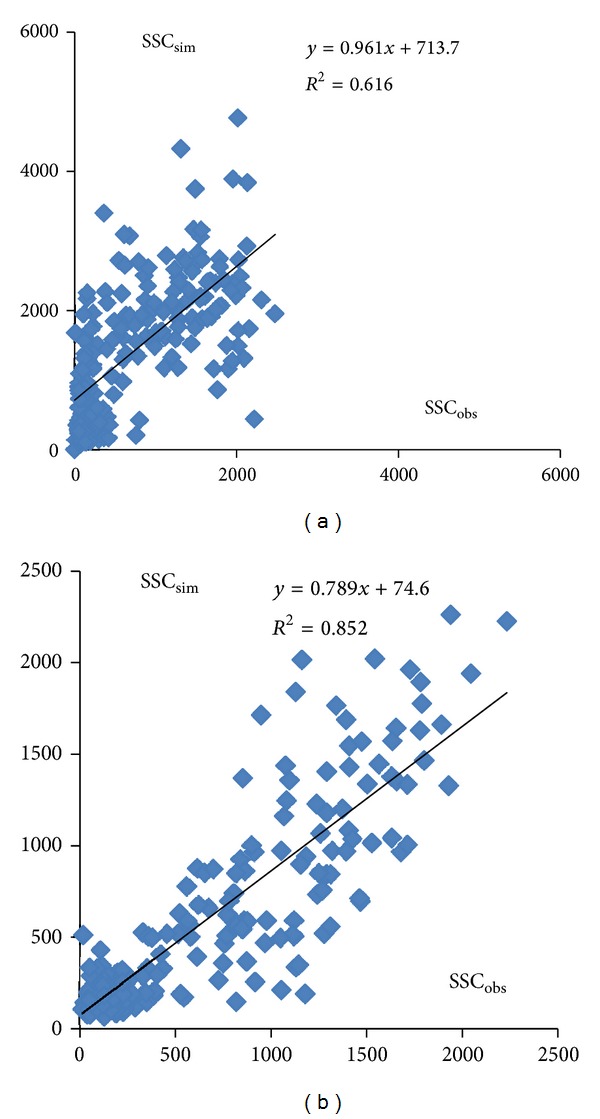
Scatterplot of observed and simulated monthly SSC from SWAT (a) and new sediment rating curve (b) in the Laichau station.

Limited by the period of rainfall data, period from 1988 to 1993 was selected as the comparison period. In order to compare the new sediment rating curve with SWAT model, SWAT model was calibrated and validated to prove its applicability in the DRB. In order to simulate sediment, streamflow should be firstly calibrated. As shown in Table 4, three statistics to evaluate the SWAT model mentioned above give agreement results. For example, the high NSE presents better results with the value of greater than 0.85 which indicate that SWAT model is reasonable to simulate streamflow. In addition, from the viewpoint of comparison between the simulated and observed monthly streamflow, results indicate that the simulated streamflow by using SWAT model has a good match with the observed values at Laichau station, as showed in Figure 6. After this, the sediment concentration was calibrated by SWAT model at Laichau station. As shown in Table 4, three statistics indicated that SWAT model could simulate the SSC reasonably. Then, we compared new sediment rating curve simulation result with SWAT model simulation result, as showed in Figure 7. The coefficient of determination of SWAT model simulation is lower than new sediment rating curve results. In addition, SWAT model trended to overpredict SSC very much in the DRB (Table 4). Based on the above, new sediment rating could simulate SSC better than SWAT model in DRB.

### 3.5. Validation in Other River Basins

**Figure 8:**
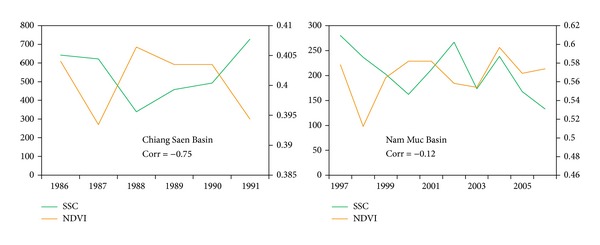
Relationship between annual SSC and NDVI (Corr: correlation coefficient).

To further confirm the performance of the best new sediment rating curve, we select another two basins to validate it. Similarly, inverse relationship between NDVI and SSC is also found out in other basins even though the correlation coefficient between SSC and NDVI is different (Figure 8).

Simulation results comparison from new sediment rating curve and common sediment rating curve in another two basins is listed in Tables 5 and 6. Not only the coefficient of determination of new sediment rating curve is higher than the common one, but also MAE is lower than the common one, which showed that new sediment rating curve also performed better than common one in these two basins. All simulation results further confirmed that vegetation cover information (NDVI) can improve the sediment rating curve and new sediment rating curve could simulate better in these East-South Asian basins.

### 3.6. Discussions

The results above showed that vegetation cover information could improve simulation results. However, the improvement is different in different basins. Summary of improvement was listed in Table 7, which showed that improvement of correlation coefficient in basin with large area was higher than small catchment. For large basins, more vegetation cover information could be obtained, which may improve the simulation results better. As a result, more accurate and high precision vegetation cover information seems more useful to improve simulation result. Actually, several previous studies have already been carried out to calculate the sediment load in terms of vegetation cover change. For example, Guzman et al. [20] tried to develop different sediment rating curves based on normalization of fractional cropland for each part of the rainy season (early, middle, and late) in three watersheds, which indicated that vegetation cover in different seasons has different effects.

Because the new sediment rating curve only has three parameters and three kinds of inputs could be applied to simulate sediment yield for basins without enough dataset, the most important point of this new sediment rating curve is not only to improve the common sediment rating curve, but also to describe the relationship among vegetation cover, runoff, and sediment load. Hence the new sediment rating curve has its potential application related to vegetation cover change. For example, in order to evaluate reservoir sediment trapping, we have to know actual reservoir sediment outflow and potential sediment outflow without considering dam effect. According to many researches, the common sediment rating curve was used to predict the potential sediment flow. Unfortunately, the potential sediment flow from common sediment rating curve does not consider vegetation cover change effects because the common curve does not consider vegetation cover change effect. As a result, the reservoir sediment trapping result does not look so reasonable. Another potential application is to evaluate vegetation cover change impacts on sediment yield change [11].

Although our new sediment rating curve has already been proved to be better than the common one, we still could not conclude that it is universal for all the basins worldwide. More validation should be carried out in basins with different area, vegetation cover types, and climate conditions in the future.

## 4. Conclusions 

In this study, we successfully considered vegetation cover information in sediment simulation and developed new sediment rating curve in Da River Basin. Main conclusions are as follows.

1. The common sediment rating curve tends to overpredict the high and underpredict the low SSC in all the three basins.
2. Vegetation cover (NDVI) can improve the sediment rating curve. Among the three new sediment rating curves, the third one is the best sediment rating curve for our research basin.
3. The new sediment rating curve can simulate better in these three Asian basins, but, for different scale basin, vegetation cover (NDVI) improved the sediment rating curve differently.
4. The most important point of this new equation is not only to improve the common sediment rating curve, but also to describe the relationship among vegetation cover, runoff, and sediment load.

## Figures and Tables

**Table 1:** Basic characters of our selected basins.

Basins	*Q* and SSC time span	Area (km^2^)	Characters
Da River Basin	1988–2004 monthly	55000	No big dam in the upstream before 2004 Main vegetation cover: forest
Chiang Sean Basin	1986–1991 monthly	185,000	No big dam before 1992 Main vegetation cover: grass and forest
Nam Muc Basin	1997–2006 monthly	2,200	No big dam existed Main vegetation cover: forest

**Table 2:** Statistics analysis between monthly observed SSC and simulated SSC from common sediment rating curve in DRB.

SSC (mg/L)	Obs	Simu	MAE	
High value	1265	1313	48	Overpredict
Low value	416	296	−120	Underpredict
Average	629	551	−78	Underpredict

Obs: monthly observed SSC; Simu: simulated SSC from common sediment rating curve.

**Table 3:** Comparison of simulation results from three new sediment rating curves and common sediment rating curve in DRB.

SSC (Mg/L)	Obs	Common		Trial A		Trial B		Trial C	
		Sim	MAE	Sim	MAE	Sim	MAE	Sim	MAE
High value	1265	1313	48	1283	18	1306	41	1274	9
Low value	416	296	−120	325	−91	328	−88	343	−73
Total average	629	551	−78	564	−65	572	−57	580	−49
*R* ^2^		0.821		0.845		0.847		0.852	

High value: SSC from July to September. Low value: SSC from remaining months.

**Table 4:** Evaluation of model simulation during the prechange period for the catchments controlled by Laichau and Tabu stations in the DRB.

	Streamflow		Sediment	
	Calibration	Validation	Calibration	Validation
*R* ^2^	0.91	0.87	0.72	0.70
NSE	0.89	0.85	0.65	0.56
PBIAS (%)	0.331	0.398	12.3	16.8

**Table 5:** Comparison of simulation results from new sediment rating curve and common sediment rating curve in Chiang Saen Basin.

SSC (Mg/L) (Chiang Saen)	Obs	Common		New one (Trial c)	
		Sim	MAE	Sim	MAE
High value	927	1030	103	1011	84
Low value	312	244	−68	252	−60
Average	517	506	−11	508	−9
*R* ^2^		0.80		0.86	

High value: SSC from July to September. Low value: SSC from remaining months.

**Table 6:** Comparison of simulation results from new sediment rating curve and common sediment rating curve in Nam Muc basin.

SSC (Mg/L) (Nam Muc)	Obs	Common		New one (Trial c)	
		Sim	MAE	Sim	MAE
High value	401	410	9	405	4
Low value	110	76	−68	102	−8
Average	207	190	−17	204	−3
*R* ^2^		0.74		0.75	

High value: SSC from July to September; Low value: SSC from remaining months.

**Table 7:** Summary of improvement compared with common rating curve in three basins.

Basin	Area (10^3^ km^2^)	*R* ^2^		
		Common	New	Improvement
Chiang Saen	185	0.80	0.86	6%
Laichau	52	0.82	0.85	3%
Nam Muc	2.2	0.74	0.75	1%
